# Triangulating evidence from longitudinal and Mendelian randomization studies of metabolomic biomarkers for type 2 diabetes

**DOI:** 10.1038/s41598-021-85684-7

**Published:** 2021-03-18

**Authors:** Eleonora Porcu, Federica Gilardi, Liza Darrous, Loic Yengo, Nasim Bararpour, Marie Gasser, Pedro Marques-Vidal, Philippe Froguel, Gerard Waeber, Aurelien Thomas, Zoltán Kutalik

**Affiliations:** 1 Center for Integrative Genomics, University of Lausanne, Lausanne, Switzerland; 2 Swiss Institute of Bioinformatics, Lausanne, Switzerland; 3 Unit of Forensic Toxicology and Chemistry, CURML, Lausanne University Hospital and Geneva University Hospitals, Geneva, Switzerland; 4 Faculty Unit of Toxicology, CURML, Faculty of Biology and Medicine, University of Lausanne, Lausanne, Switzerland; 5 Center for Primary Care and Public Health, University of Lausanne, Lausanne, Switzerland; 6 Institute for Molecular Bioscience, The University of Queensland, Brisbane, Australia; 7 Department of Medicine, Internal Medicine, Lausanne University Hospital and University of Lausanne, Lausanne, Switzerland; 8 Inserm UMR1283, CNRS UMR8199, European Genomic Institute for Diabetes (EGID), Université de Lille, Institut Pasteur de Lille, Lille University Hospital, Lille, France; 9 Department of Metabolism, Imperial College London, London, UK

**Keywords:** Metabolic disorders, Genetics

## Abstract

The number of people affected by Type 2 diabetes mellitus (T2DM) is close to half a billion and is on a sharp rise, representing a major and growing public health burden. Given its mild initial symptoms, T2DM is often diagnosed several years after its onset, leaving half of diabetic individuals undiagnosed. While several classical clinical and genetic biomarkers have been identified, improving early diagnosis by exploring other kinds of omics data remains crucial. In this study, we have combined longitudinal data from two population-based cohorts CoLaus and DESIR (comprising in total 493 incident cases vs. 1360 controls) to identify new or confirm previously implicated metabolomic biomarkers predicting T2DM incidence more than 5 years ahead of clinical diagnosis. Our longitudinal data have shown robust evidence for valine, leucine, carnitine and glutamic acid being predictive of future conversion to T2DM. We confirmed the causality of such association for leucine by 2-sample Mendelian randomisation (MR) based on independent data. Our MR approach further identified new metabolites potentially playing a causal role on T2D, including betaine, lysine and mannose. Interestingly, for valine and leucine a strong reverse causal effect was detected, indicating that the genetic predisposition to T2DM may trigger early changes of these metabolites, which appear well-before any clinical symptoms. In addition, our study revealed a reverse causal effect of metabolites such as glutamic acid and alanine. Collectively, these findings indicate that molecular traits linked to the genetic basis of T2DM may be particularly promising early biomarkers.

## Introduction

Type 2 Diabetes Mellitus (T2DM) is a major public health concern and its prevalence is increasing. Almost 500 million individuals are currently affected worldwide by diabetes and almost 700 million may be affected by 2045 [https://www.diabetesatlas.org/en/sections/worldwide-toll-of-diabetes.html]. Diabetes remains among the leading causes of cardiovascular disease, blindness, kidney failure, and lower-limb amputation. By the time T2DM is diagnosed, many individuals have already established end-organ damage including neuropathy, kidney failure and/or premature cardiac or brain atherosclerosis. Diabetes and pre-diabetes are diagnosed by routinely assessed clinical markers [glycaemia and glycated haemoglobin (HbA_1_c) levels] above a given threshold. Still, agreement between the different markers in diagnosing T2DM is not optimal^1^, and their screening capacity for pre-diabetes is low^2^. While these markers are powerful predictors of the disease, they are far from perfect for the identification of individuals who are prone to develop T2DM. Early detection of individuals with high T2DM predisposition is important as non-pharmacological approaches (i.e. lifestyle changes) can reduce substantially (and at a reduced cost) the risk of developing T2DM^3,4^. Several predictive scores have been developed using clinical and genetic data (e.g.^5,7^.), but their performance is far from optimal. Since many metabolites and proteins are expected to be altered in pre-diabetic state, using different omics profiles in addition to the classical clinical, biological and genetic risk factors is expected to increase the prediction accuracy.

Several cross-sectional and longitudinal metabolomic studies, focussing on blood samples using targeted approaches, have been initiated to identify candidate biomarkers of pre-diabetes, with a few exceptions employing untargeted approaches (e.g. a cross-sectional study of 115 T2DM individuals^8^). In the population-based cooperative health research of Augsburg (KORA), 140 metabolites were quantified for 4297 participants and several metabolites altered in pre-diabetic individuals have been identified^9^. Using metabolite-protein network and targeted approaches on serum samples, Wang-Sattler et al. identified seven T2DM-related genes associated with these metabolites by multiple interactions with four enzymes. Lysophosphatidylcholine (18:2) and glycine were strong predictors of glucose intolerance, even 7 years before disease onset. These metabolites, in addition to sugar metabolites, acylcarnitines and other aminoacids, have been identified as predictors of T2DM also in the European Prospective Investigation into Cancer and nutrition cohort^10^. More recently, Padberg et al*.* described a metabolic signature that includes glyoxylate associated with T2DM and prediabetic individuals^11^. Wang et al. using longitudinal data on 201 incident T2DM cases, identified a signature of five branched-chain and aromatic metabolites for which individuals in the top quartile exhibited a five-fold higher risk to develop T2DM^12^. Particularly, a combination of three amino acids predicted future T2DM, with a more than five-fold higher risk for individuals in top quartile, suggesting that amino acid profiles could aid in diabetes risk assessment. These results were confirmed by a recent meta-analysis from 8 prospective studies on 8000 individuals, which found a higher risk of T2DM for isoleucine, leucine, valine and phenylalanine^13^.

By targeting serum carnitine metabolites on 173 incident T2DM cases among 2519 patients with coronary artery disease, Strand et al*.* demonstrated that trimethyl-lysine, g-butyrobetaine, as both precursors on free carnitine and palmitoyl-carnitine, predict long-term risk of T2DM independently of traditional risk factors^14^.

As another example, shotgun lipidomics was applied in a transversal study on plasma of pre-diabetic mice from different genetic backgrounds and revealed a group of ceramides correlated with glucose tolerance and insulin secretion^15^. These results were interestingly confirmed by quantitative analysis in the plasma of individuals from two population-based prospective cohorts showing that dihydro-ceramides were significantly elevated in the plasma of individuals who will progress to diabetes up to 9 years before disease onset^15^. Other studies have struggled to identify the contribution of individual metabolites and focused more on metabolome-wide prediction^16^, which are difficult to replicate.

The previously listed studies provide several important candidate metabolites to benchmark our experimental and modelling setup. Here, we used a subset of the CoLaus study intentionally enriched for T2DM incident cases to maximise discovery power of baseline metabolite levels being associated with developing T2DM at a later follow-up stage. We compared our findings with a similarly sized population-based cohort, DESIR, and also with bidirectional Mendelian randomisation using metabolite- and T2DM QTLs as instruments.

## Methods

### The CoLaus study

**Figure 1:**
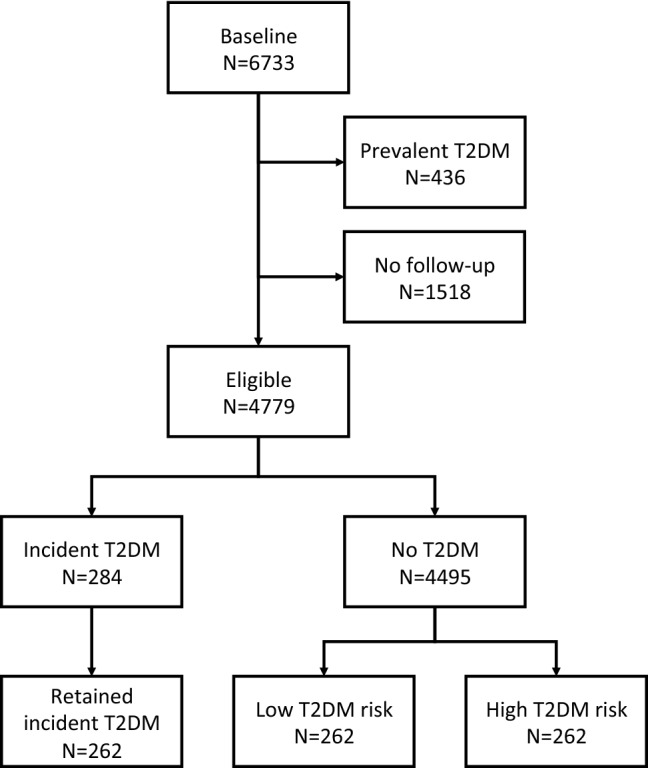
Flowchart of participant selection from CoLaus study.

The CoLaus study (www.CoLaus-psycolaus.ch) is a population-based prospective study based on a single random sample of 6733 participants from the overall population aged between 35 and 75 living in Lausanne (10). The baseline survey was conducted between 2003 and 2006. Each participant was extensively phenotyped regarding personal, lifestyle and cardiovascular risk factors; extensive blood and urine characterization was performed, and over 500,000 SNPs were directly genotyped and a further 20.4 million imputed (with r2-hat > 0.3). The first follow-up was performed between April 2009 and September 2012; median follow-up time was 5.4 (average 5.6, range 4.5–8.8) years; it included 5064 participants, and the 5.5-year incidence of T2DM was 6.5%, with 284 incident cases. The second follow-up was performed between May 2014 and April 2017; median follow-up was 10.7 (average 10.9, range 8.8–13.6) years. In this study, we selected 262 T2DM incident cases at the first follow-up and 524 controls matched for sex, age and baseline glucose. For each case, two types of controls were selected: one with a very low risk of T2DM (as assessed by a multivariable T2DM risk score^17^) and one with pre-diabetes (with a high-risk score, but no T2DM at the CoLaus second follow-up) (Fig. 1). Incident T2DM cases were defined as fasting glucose ≥ 7 mmol/L and/or presence of antidiabetic drug treatment and/or HbA1c ≥ 6.5%. The most important study characteristics are included in Table 1. All research was performed in accordance with relevant guidelines and regulations. The study protocols were approved by the Ethical Committee of the Canton de Vaud and all participants provided written informed consent.

**Table 1:** Sample characteristics of the CoLaus and DESIR studies.

	CoLaus			DESIR			
	Y0	Y5	Y10	Y0	Y3	Y6	Y9
N	788	788	673	984	961	932	957
Gender (woman)	268 (34.0)	268 (34.0)	240 (35.7)	469 (47.7)	455 (47.3)	442 (47.4)	462 (48.3)
Age (years)	56.2 ± 9.7	61.8 ± 9.7	66.7 ± 9.5	48.2 ± 10.1	51.2 ± 10.1	54.2 ± 10.1	57.2 ± 10.1
**Smoking categories (%)**							
Never	283 (35.9)	280 (36.0)	237 (38.4)	484 (49.2)	474 (49.3)	456 (48.9)	473 (49.4)
Former	302 (38.3)	339 (43.6)	284 (46.0)	278 (28.3)	272 (28.3)	264 (28.3)	270 (28.2)
Current	203 (25.8)	159 (20.4)	96 (15.6)	222 (22.6)	215 (22.4)	212 (22.7)	214 (22.4)
Alcohol consumption (g/week)	5 [1–11]	4 [0–10]	4 [0–10]	2.51 [0.14—3.29]	2.54 [0.29—4.04]	2.48 [0.29—4.04]	2.29 [0.29—3.52]
Body mass index (kg/m^2^)	27.1 ± 4.6	27.5 ± 4.9	27.8 ± 5.1	25.1 ± 4.0	25.6 ± 4.4	26.0 ± 4.5	26.2 ± 4.5
Waist (cm)	94.2 ± 13.1	97.1 ± 13.2	97.7 ± 13.9	84.5 ± 12.1	85.9 ± 12.6	87.5 ± 12.9	88.2 ± 13.1
HDL (mmol/L)	1.53 ± 0.42	1.53 ± 0.44	1.49 ± 0.46	1.61 ± 0.42	1.51 ± 0.39	1.62 ± 0.40	1.47 ± 0.34
Triglycerides (mmol/L)	1.3 [0.9–1.9]	1.3 [0.9–1.8]	1.2 [0.9–1.7]	1.2 [0.7—1.5]	1.3 [0.7—1.6]	1.3 [0.8—1.6]	1.3 [0.8—1.5]
Glucose (mmol/L)	5.68 ± 0.66	6.38 ± 1.13	5.98 ± 1.39	5.39 ± 0.57	5.53 ± 0.84	5.63 ± 1.04	5.67 ± 1.06
Insulin (μU/mL)	8.2 [5.1–12.4]	8.3 [5.2–13.5]	9.8 [5.9–14.8]	7.1 [4.2—8.3]	7.0 [4.2—8.4]	7.5 [3.9—9.0]	7.3 [4.1—8.7]
HOMA2-IR	2.1 [1.3–3.3]	2.4 [1.4–4.0]	2.6 [1.4–4.2]	1.1 [0.7—1.3]	1.1 [0.7—1.3]	1.2 [0.7—1.5]	1.2 [0.7—1.4]

Yn denotes n years after baseline (e.g. Y3 means a follow-up 3 years after baseline). N stands for sample size. Values are either number (% of total), median [interquartile range] or mean ± standard deviation.

### The DESIR cohort

The prospective D.E.S.I.R. cohort is a 9-year follow up study of 2391 middle-aged European ancestry participants^18–20^. We analysed participants from a case-cohort design embedded within the larger cohort that includes 231 cases of incident T2DM and 836 participants randomly sampled from the entire cohort. Baseline and follow-up clinical characteristics of participants included in the training population are shown in^5^ (see Table 1). T2DM was defined using one of the following criteria: use of glucose lowering medication, fasting plasma glucose [FG] ≥ 7 mmol/L, or glycated hemoglobin A1c [HbA_1c_] ≥ 6.5% (48 mmol/mol). Clinical and biological evaluations were performed at inclusion and after 3, 6, and 9 years, as previously described^21^. All research was performed in accordance with relevant guidelines and regulations. All participants provided written informed consent and the study protocol was approved by the Ethics Committee for the Protection of Subjects for Biomedical Research of Bicêtre Hospital, France. Metabolites measurements have been described elsewhere in full details^21^.

### Targeted metabolomics analysis

Plasma and urine samples collected at the baseline of the CoLaus cohort were processed for targeted metabolomics analysis as described elsewhere^22^. Briefly, metabolites were extracted from 100 µL of plasma or urine samples and Quality Control (QC) samples using a cold methanol-ethanol solvent mixture in a 1:1 ratio. After centrifugation at 14,000 rpm for 15 min, supernatant was recovered, evaporated and resuspended in 100 µL (for plasma) or 200 µL (for urine) of H_2_O:MeOH (9:1). 5 µL of the samples were analyzed by LC-MRM/MS on a hybrid triple quadrupole-linear ion trap QqQ_LIT_ (Qtrap 5500, Sciex) hyphenated to a LC Dionex Ultimate 3000 (Dionex, Thermo Scientific). Analyses were performed in positive and negative electrospray ionization using a TurboV ion source. The chromatographic separation was performed on a Kinetex column C18 (100 × 2.1 mm, 2.6 µm). The mobile phases were constituted by A: H_2_O with 0.1% FA and B: ACN with 0.1% FA for the positive mode. In the negative mode, the mobile phases were constituted by A: ammonium fluoride 0.5 mM in H_2_O and B: ammonium fluoride 0.5 mM in ACN. The linear gradient program was 0–1.5 min 2%B, 1.5–15 min up to 98%B, 15–17 min held at 98% B, 17.5 min down to 2%B at a flow rate of 250 µL/min.

The MRM/MS method included 299 and 284 transitions in positive and negative mode respectively, corresponding to 583 endogenous metabolites. For each biological matrix, the 786 samples were prepared and analyzed in 8 batches. In order to monitor the signal drift and system performance over time, and to avoid repeated thawing-freezing cycles of the study samples, quality control (QCs) surrogate samples were used. These QC samples were prepared in the same way and at the same time of the study samples from aliquots of a pool of human plasma or urine that was the same for all the analytical batches. QC samples were injected every 8 samples in both positive and negative modes.

The MS instrument was controlled by Analyst software v.1.6.2 (AB Sciex). Peak integration was performed with MultiQuant software v.3.0 (AB Sciex). The integration algorithm was MQ4 with a Gaussian smoothing of a half-width equal to 1.5 points. For plasma samples, the analysis was narrowed to the 124 and 48 metabolites that were detected in all samples with a noise percentage of 80% and a gaussian peak shape, in positive and negative modes, respectively. For urine samples, we detected 124 and 77 metabolites in positive and negative modes, respectively. In case of remaining missing values, they were replaced by the lowest value of the corresponding metabolite. To correct for batch effect, raw data were normalized with the dbnorm package^23^, by using the *ber* model.

### Statistical approaches

We performed logistic- and linear regression analysis to test for association between baseline metabolite levels and T2DM incidence and glucose level changes, respectively. We included the following covariates: family history of diabetes, smoking status, body-mass index (BMI), HDL cholesterol, triglycerides, insulin, glucose and HOMA measure at baseline. Since none of our association *P* values passed strict Bonferroni correction for multiple testing (*P* < 0.05/172) or FDR correction (*P*_FDRadj_ < 0.05), we declared *P* values below 0.01 as suggestively significant.

### Bi-directional metabolome-wide mendelian randomization

To explore the causal paths between metabolites and glucose and T2DM, we performed Mendelian randomization (MR), an instrumental variable method to distinguish correlation from causation in observational data^24^. The idea of MR is to use genetic variants as instrumental variables to attempt causal inference about the effect of modifiable risk factors, which can overcome some types of confounding and reverse causation.

We performed two-sample bidirectional MR. We tested whether genetically varying levels of a particular metabolite affect the risk for elevated glucose and T2DM (we call this MR) and whether genetically increased risk of T2DM or elevated glucose is associated with circulating levels of a particular metabolite (we call this reverse MR). The associations between the instrumental variables and the exposure and the outcome are estimated from independent studies.

To run MR for each metabolite, as instrumental variables, we used independent (pairwise r^2^ < 0.01) significant (*P* < 1 × 10^–05^) SNPs associated with the metabolite in study (Supplementary Table 1). Such data are from a large GWAS performed for 453 whole blood metabolites in 7824 European individuals^25^. To run the reverse MR, for glucose and diabetes we used as instrumental variables the independent (r^2^ < 0.01) genome-wide significant SNPs (P < 5 × 10^–08^) found by the GWASs performed on UKBB and the DIAGRAM Consortium for T2DM^26^, respectively (Supplementary Table 2).

## Results

### Study characteristics

Selected basic features of the CoLaus study are listed in Table 1. We selected 788 participants, including 263 T2DM incident cases at the first follow-up and 525 controls matched for sex, age and baseline glucose. Summary data are expressed either as counts (and percentage) for categorical variables and as median [interquartile range] or mean ± standard deviation for continuous variables.

### Metabolites association scan

Based on quality criteria such as sensitivity and peak shape, we detected 172 urine and plasma metabolites (MS) in the 788 selected CoLaus participants. The analytical samples were collected at the baseline and included 525 participants without diabetes and 263 participants who became diabetic over the following 10 years. For each metabolite, we ran logistic/linear regression analysis with diabetes incidence/change in glucose levels as outcome and family history of diabetes, smoking status, body-mass index (BMI), HDL cholesterol, triglycerides, insulin, glucose and HOMA measure at baseline, metabolite levels as independent variables. Note that we tested only one metabolite at a time and ran a separate model for each metabolite.

**Figure 2:**
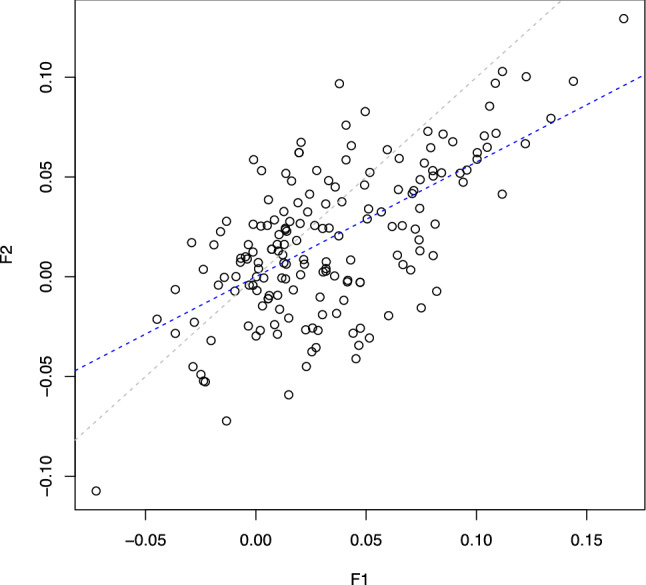
Linear relationship between the effects estimated in the first (F1) and second (F2) follow-up. The blue and grey lines represent the regression and the identity line respectively.

Here, all the results are based on the first CoLaus follow-up which is the best powered for predictive analysis. Indeed, when we compared the effects estimated in the first and second follow-up we observed significant weaker effects in the second follow-up (*P*_t-test_ = 1.38 × 10^–05^, see Fig. 2). It is logical that as more time passes, other factors emerge that may influence diabetes incidence, reducing the predictive power of baseline biomarkers.

The metabolome-wide association scan revealed seven metabolites associated with glucose change at suggestively significant level (*P* < 0.01) in the CoLaus study, see Table 2. When we meta-analysed metabolome-wide results from the CoLaus and DESIR studies, we similarly found leucine and four additional suggestively significant (*P* < 0.01) metabolites associated with glucose change (Table 3). As DESIR is a 9-year follow up study where biological evaluations were performed every 3 years, we used the data collected in the second follow-up (after 6 years) to match as closely as possible the 5-years follow-up performed in CoLaus.

**Table 2:** Metabolites associated with glucose change in the CoLaus cohort.

Metabolite	Colaus		DESIR		CoLaus + DESIR	Metabolite → glucose MR		Glucose → metabolite MR		Metabolite → T2DM MR		T2DM → metabolite MR	
	Effect	*P* value	Effect	*P* value	*P* value	Effect	*P* value	Effect	*P* value	Effect	*P* value	Effect	*P* value
Valine (HMDB0000883)	0.095	9.00E−04	0.005	0.853	0.069	0.000	1.000	0.104	0.023	0.023	0.008	0.115	0.003
Leucine (HMDB0000687)	0.088	0.0021	0.046	0.158	2.90E−03	0.002	0.372	0.042	0.360	0.007	0.011	0.159	5.79E−05
Sarcosine (HMDB0000271)	0.081	0.0039	0.014	0.581	0.076	x	x	x	x	x	x	x	x
4-Hydroxyproline (HMDB0006055)	0.083	0.0042	− 0.039	0.116	0.94	x	x	x	x	x	x	x	x
Glutamic acid (HMDB00148)	0.074	0.009	− 0.038	0.185	0.656	0.01	0.147	0.069	0.134	0.012	0.12	0.18	2.84E−06
Homoserine (HMDB0000719)	0.078	0.0094	x	x	x	x	x	x	x	x	x	x	x

For each metabolite we report its effect size on glucose in the CoLaus and DESIR cohorts, the combined *P* value and the forward and reverse causal effect on glucose and T2DM estimated by Mendelian Randomisation. “X” indicates missing value, i.e. when the metabolite was not available for the respective analysis. The ID for the Human Metabolome Database is indicated for each metabolite.

**Table 3:** Additional metabolites found significantly associated with glucose change after combining CoLaus and DESIR Cohort results.

Metabolite	Colaus		DESIR		CoLaus + DESIR	Metabolite → glucose MR		Glucose → metabolite MR		Metabolite → T2DM MR		T2DM → metabolite MR	
	Effect	*P* value	Effect	*P* value	*P* value	Effect	*P* value	Effect	*P* value	Effect	*P* value	Effect	*P* value
L-carnitine (HMDB00062)	0.046	0.111	0.091	3.26E−04	8.30E−05	0.00	0.358	0.05	0.314	0.00	0.199	0.09	0.027
Leucine (HMDB0000687)	0.088	0.002	0.046	0.158	0.003	0.00	0.372	0.04	0.360	0.01	0.011	0.23	5.81E−07
Phenylacetylglutamine (HMDB0006344)	− 0.040	0.204	− 0.058	0.013	0.006	− 0.001	0.429	0.037	0.415	− 0.01	0.2	0.008	0.836
Pantothenic acid (HMDB00210)	0.047	0.114	0.065	0.026	0.007	x	x	x	x	x	x	x	x
Cortisol (HMDB00063)	− 0.049	0.061	− 0.046	0.059	0.009	− 0.01	0.116	− 0.02	0.669	− 0.01	0.198	− 0.07	0.086

For each metabolite we report its effect size on glucose in CoLaus and DESIR cohorts, the combined *P* value and the forward and reverse causal effect on glucose and T2DM estimated by Mendelian Randomisation. The ID for the Human Metabolome Database is indicated for each metabolite.

### Metabolome-wide mendelian randomization

Table 4 shows the significant results from the metabolome-wide MR approach. Among the 453 testable metabolites, genetically altered levels of one and six metabolites were found significantly associated with glucose and T2DM, respectively. These include betaine, mannose, lysine, and three phospholipid species.

**Table 4:** Metabolome-wide mendelian randomization results.

	Metabolite	Metabolome-wide mendelian randomization results		
		beta	se	*P*
Metabolite → Glucose	Betaine (HMDB0000043)	− 0.022	0.005	4.89E−05
Metabolite → T2DM	Mannose (HMDB0000169)	0.052	0.007	9.06E−15
	Lysine (HMDB0000182)	0.047	0.009	5.43E−08
	X-02249*	− 0.033	0.008	3.98E−05
	LysoPE(18:2(9Z,12Z)/0:0) (HMDB0011507)	− 0.042	0.008	1.78E−07
	LysoPC(14:0/0:0) (HMDB0010379)	− 0.048	0.012	3.52E−05
	LysoPE(18:1(9Z)/0:0) (HMDB0011506)	− 0.050	0.011	1.63E−06
Glucose → Metabolite	Fructose (HMDB0000660)	0.257	0.065	6.60E−05
	Mannose (HMDB0000169)	0.404	0.065	5.26E−10
	Glucose (HMDB0000122)	0.698	0.065	9.29E−27
	Hyodeoxycholic acid (HMDB0000733)	0.280	0.066	2.35E−05
T2DM → metabolite	Leucine (HMDB0000687)	0.232	0.046	5.81E−07
	2-hydroxypropanoic acid (HMDB0144295)	0.186	0.046	5.79E−05
	Fructose (HMDB0000660)	0.194	0.045	1.54E−05
	Mannose (HMDB0000169)	0.436	0.049	7.68E−19
	Proline (HMDB0000162)	0.190	0.045	2.73E−05
	Glucose (HMDB0000122)	0.380	0.047	4.29E−16
	Glutamic acid (HMDB0000148)	0.213	0.045	2.28E−06
	Alanine (HMDB0000161)	0.191	0.046	3.38E−05
	X-11317*	− 0.183	0.046	7.98E−05
	X-12696*	− 0.185	0.045	4.79E−05
	X-12844*	0.180	0.045	6.63E−05
	X-14625*	0.232	0.045	3.26E−07

The ID for the human metabolome database is indicated for each metabolite.

*Indicates metabolites that are not identifiable in the existing databases.

Applying the reverse MR, we found that the genetic predisposition to T2DM is associated with the levels of 12 metabolites.

None of the metabolites found significant by MR was associated with glucose in CoLaus (*P* > 0.05). By contrast, among the seven metabolites reported in Tables 2 and 3, three were testable with MR. While we did not observe any significant effect for glucose, T2DM showed a significant causal effect on valine (*P* = 0.003), leucine (*P* = 5.8 × 10^–05^) and glutamate (*P* = 2.8 × 10^–06^).

## Discussion

Using mass-spectrometry targeted metabolomics analysis, we identified a panel of metabolites whose levels are associated with glucose changes before the onset of T2DM in the CoLaus Cohort. We replicated our findings in an independent study (DESIR), which reassuringly revealed five metabolites with combined *P* value below 0.01, including l-carnitine, leucine, and cortisol. In addition, we applied a metabolome-wide Mendelian Randomization (MR) approach which allowed us to confirm the causal effect of leucine on T2DM, but also to identify new reverse causal relationships between glucose/T2DM and metabolites, such as leucine, valine, glutamic acid, alanine and mannose.

While we focused our analysis on predicting T2DM for a 5-years follow-up period, we observed that the effect of the potentially predictive metabolites diminished over time. This is unsurprising as risk factors change over time, hence more and more unknowns contributing to diabetes conversion accumulate with time. We have noticed, furthermore, that more than 80% (140/172) of the metabolite effects are positive, meaning that generally an increased level of metabolites represents a risk factor for diabetes. This observation needs to be considered with caution, since it might be due to a latent diabetes-associated confounding factor, which is linked to overall metabolite concentration. This explanation is rather unlikely since we accounted for metabolomic principal components in all association scan.

The confirmed metabolites have been repeatedly supported by various types of evidence in both human and model organisms. Individuals with obesity and T2DM have elevated levels of branched-chain amino acids (leucine, isoleucine and valine)^12,27–30^. Such changes are already present before the onset of diabetes^12,13,31,32^ and their causal role is believed to be exerted via the modulation of the mTOR pathway. Increased leucine levels can lead to insulin resistance via activation of the TORC1 pathway, with induction of beta cell proliferation and insulin secretion^12,33^ and disruption of insulin signal in skeletal muscle^34^. On the other hand, insulin resistance enhances protein catabolism in skeletal muscle, which can increase the release of branched-chain amino acids^35^. Moreover, hyperglycemia negatively correlates with adipose tissue expression of genes involved in branched-chain amino acid oxidation, which can further contribute to raise the levels of BCAA^35^. Thus, so far it remains unclear whether the observed BCAA changes are only a consequence of hyperglycemia or if they have a causative role in the development of T2DM.

Another metabolite which is displaying a significant association with glucose changes in the CoLaus cohort is glutamic acid, although this is not replicated in the DESIR study. A link between increased glutamate levels and insulin resistance traits was already observed in multiple cohorts^28,32,36^, and more recently, a meta-analysis conducted in 18 prospective studies highlighted glutamate as positively correlated with T2DM^37^. Glutamate is a glucogenic aminoacid, which can enter into the Krebs cycle through its conversion to α-ketoglutarate. In addition, it can favour gluconeogenesis by increasing the transamination of pyruvate to alanine^38^, and can directly stimulate glucagon release from pancreatic α-cells^39^. However, its possible causal role remains controversial and several reports suggest that it is rather a reduced ratio between glutamine, a glutamate derivative, and glutamate itself, that is informative of metabolic risk^36,40^.

In our study, free carnitine, cortisol, phenylacetylglutamine and pantothenic acid also appeared as significantly associated to the development of T2DM, although only when our data were combined with the French cohort (CoLaus + DESIR). Carnitine esterification with fatty acids is required for the shuttling of the latter into the mitochondria for fatty acid oxidation. Lower levels of free carnitine were reported in diabetic individuals^14,41,42^, while changes of acylcarnitines and/or carnitine precursors were highlighted in several studies as indicators of prediabetes/T2DM^9,10,29^, although data are not always consistent. Our targeted LC–MS method included several of these acylcarnitines, such as acetylcarnitine, propionylcarnitine, and isovalerylcarnitine, but no significant association was found with subsequent development of T2DM.

Cortisol dysregulation has been linked to T2DM in cross-sectional and longitudinal studies^43,44^. More specifically, diabetic individuals present a flattened diurnal cortisol curve compared to non-diabetic ones^45^, with lower morning and higher afternoon and evening concentrations^46^. Interestingly, high levels of evening cortisol were also shown to be predictive of T2DM development in an occupational cohort^47^. Even though the mechanisms underlying this association are not completely understood, cortisol contributes to many metabolic processes that can potentially perturb glucose homeostasis^48^. Cortisol has a major role in raising glucose levels through gluconeogenesis activation. Moreover, it induces lipolysis, thus increasing the release of free fatty acids that may favour the impairment of glucose uptake. Of note, diabetes is considered a common complication in clinical states characterized by prolonged hypercortisolaemia, such as in Cushing disease^49^. However, the causality of this association remains to be determined. Phenylacetylglutamine is a nitrogenous metabolite almost exclusively derived from the gut microbiota conversion of phenylalanine^50^. Its accumulation is known to occur in uraemia and was shown to be increased in type 2 diabetic patients, particularly in association with renal damage^51,52^. Other findings with variable degree of evidence in our study have also solid corroborating literature. Sarcosine (N-methylglycine) is an intermediate and by-product in glycine synthesis and has been found to be a moderately strong (OR = 1.3) predictor of T2DM incidence^53^. More specifically, the addition of urine sarcosine to other established predictors of incident T2DM was shown to improve model performance and T2DM risk prediction in a cohort of 4164 patients with suspected stable angina pectoris. Diabetic individuals have higher circulating proline levels and, moreover, proline-induced insulin transcription impairment may contribute to the β-cell dysfunction observed in T2DM^54^.

### Causal inference

Drawing causal inference is extremely difficult. While most human studies are observational and cross-sectional predominantly, only correlations are calculated between a disease status and the levels of various predictors. Such a simple measure cannot tease apart forward-, reverse causation or confounding. Longitudinal studies provide more specific directional link (called Granger causality) between a potential biomarker and disease outcomes. With its roots in differential equation modelling, an association between the baseline level of a predictor and the change of the outcome over time may imply a causal relationship. An orthogonal axis of evidence for causality can be provided by Mendelian randomization (MR), where exposure-associated genetic markers act as instruments to tease out the causal relationship between a potential risk factor and an outcome^24^. Interventional studies, due to their intrusive nature are performed mostly in model organisms and can help triangulating causal evidence. Comparisons between the different approaches for causality have been very scarce due to the little overlap between the respective scientific communities. A pioneering work^55^ in this aspect has shown good agreement between disease-to-biomarker MR results, observational correlation and longitudinal associations. However, their longitudinal association included exposure (adiposity) change regressed on outcome (metabolite level) change, which implies no directionality and is not the intuitive way to perform such analysis.

Several MR studies have explored the causal effect of possible metabolite markers on T2DM. However, they mainly focused on classic blood lipid markers^56–58^ and on aminoacids previously associated to T2DM, such as BCAAs^4,59,60^. Among the previously investigated metabolites we could test only 12 (2-methylbutyroylcarnitine, alanine, bilirubin, citrulline, glutamate, isoleucine, leucine, N-acetylglycine, phenylalanine, tryptophan, tyrosine and valine). Apart from replicating the association of leucine and valine, we have shown that leucine has bidirectional causal relationship with diabetes, with a larger reverse (diabetes to leucine) causal effect. Our finding is consistent with a previous report that suggested that insulin resistance might drive higher levels of circulating BCAAs^56^. Moreover, in our study, (predisposition to) diabetes has a significant causal effect also on glutamate and valine, while their direct effect on diabetes is not significant. Hence small molecules targeting these metabolites may be more effective for treating downstream organ damage of T2DM, such as cardiovascular disease, neuropathies or nephropathy. In line with this hypothesis, glutamate accumulation in the retina can cause neurotoxicity and the development of diabetic retinopathy^61,62^, even though the actual connection between plasma and retinal glutamate levels remains to be assessed^40^.

Interestingly, our metabolome-wide MR approach further highlighted new causal relationships between additional metabolites and glucose/T2DM. Betaine, for instance, which was previously found to have a protective role in T2DM^53^, appears to have a causal effect on glucose. Alterations of lysine levels were also already associated to T2DM risk^63^. Another interesting metabolite, which shows bidirectional causal effect on T2DM in our study, is mannose, a hexose with essential function for glycoprotein synthesis. Mannose was repeatedly found as associated with high glucose levels and with the development of T2DM in prospective studies^8,10,64,65^. Of note, this carbohydrate might be directly synthesized from glucose^66^, which could explain the causal effect of glucose itself and of T2DM on mannose levels, while the understanding of the opposite effect (metabolite on T2DM onset) requires more investigations. Finally, we found a significant causal effect of T2DM on alanine levels, in agreement with a recent MR report^59^. Increased levels of alanine aminotransferases (ALT), the enzymes catalysing the conversion of alanine to pyruvate and glutamate, were already associated to T2DM, and could therefore underlie the observed causal effect.

### Strengths and limitations

Our study has numerous strengths. First, is our use of two well characterized prospective cohorts (one for replication), whose participants have been followed longitudinally for more than 10 years. This approach allowed us to investigate potential biomarkers in blood samples collected when individuals were still free of diabetes. Second, the robustness of our targeted methods and of our results is evidenced by the fact that we confirm many previous findings. Third, we triangulate evidence by combining these longitudinal association results with other causal inference techniques, such as MR.

The major limitation of this study is the relatively low number of incident diabetes cases that we could analyse, which prohibited us from new discoveries with unequivocal statistical evidence. In the light of these findings, we recommend future research focussing more on untargeted metabolomic approaches better exploring the vast space of metabolite species and the investigation of other omics biomarkers in parallel.

Another limitation concerns the MR analysis: pleiotropic effects of the chosen genetic instruments may lead to biased estimates. They may be underpowered because the metabolite GWASs were performed in only 7824 individuals. In addition, the causal effect of metabolites without known QTLs cannot be investigated.

### Conclusions

Our study has confirmed most of the identified-to-date metabolites in a medium-sized longitudinal population-based study (enriched for incident cases) and provided complementary evidence from bi-directional MR. However, the quest for early metabolic biomarkers predicting the development of T2DM requires more research effort including larger studies in order to understand the potentially minute contributions of many circulating metabolites.

## Supplementary Information

Supplementary Information 1.

Supplementary Information 2.
