# Supplementary material for: Triangulating evidence from longitudinal and Mendelian randomization studies of metabolomic biomarkers for type 2 diabetes
Source: Sci Rep. 2021 Mar 18;11:6197. doi: 10.1038/s41598-021-85684-7 (PMC7973501; doi:10.1038/s41598-021-85684-7)
Supplement: Supplementary file 1 — Supplementary Information 1. [file 41598_2021_85684_MOESM1_ESM.docx]

**Triangulating evidence from longitudinal and Mendelian randomization studies of metabolomic biomarkers for type 2 diabetes**

Eleonora Porcu^1,2,+^, Federica Gilardi^3,4,+^, Liza Darrous^5,2^, Loic Yengo^6^, Nasim Bararpour^3,4^, Marie Gasser^3,4^, Pedro Marques Vidal^7^, Philippe Froguel^8,9^, Gerard Waeber^7^, Aurelien Thomas^3,4,#^, Zoltán Kutalik^5,2,#^

^1^ Center for Integrative Genomics, University of Lausanne, Lausanne, Switzerland.

^2^ Swiss Institute of Bioinformatics, Lausanne, Switzerland

^3^ Unit of Forensic Toxicology and Chemistry, CURML, Lausanne University Hospital and Geneva University Hospitals, Geneva, Switzerland

^4^ Faculty Unit of Toxicology, CURML, Faculty of Biology and Medicine, University of Lausanne, Lausanne, Switzerland

^5^ Center for Primary Care and Public Health, University of Lausanne, Lausanne, Switzerland

^6^ Institute for Molecular Bioscience, The University of Queensland; Brisbane, Australia

^7^ Department of Medicine, Internal Medicine, Lausanne University Hospital and University of Lausanne, Lausanne, Switzerland

^8^ Inserm UMR1283, CNRS UMR8199, European Genomic Institute for Diabetes (EGID), Université de Lille, Institut Pasteur de Lille, Lille University Hospital, Lille, France.

^9^ Department of Metabolism, Imperial College London, London, UK.

^+^ Co-first authors

^#^ Co-last authors

**SUPPLEMENTARY MATERIAL**

Supplementary Figure1. Scatter plot showing the metabolites effect sizes estimated before and after correcting for creatinine.
